# Supplementary material for: Baseline Characterization of the Gut Microbiota of Field and Colony Populations of Phlebotomus tobbi and Preliminary Assessment of the Anti-Leishmanial Activity of Cultivable Bacteria
Source: Pathogens. 2026 Jun 23;15(7):658. doi: 10.3390/pathogens15070658 (PMC13416292; doi:10.3390/pathogens15070658)
Supplement: Supplementary file 1 [file pathogens-15-00658-s001.zip › pathogens-4357168-File S1/Results.html]

Results


# Results

#### Zymo Research Corporation

# 1 Sample Information

The following Sample Information Table shows information for up to 10 samples. For projects with more than 10 samples, the Complete Sample Information Table can be accessed by clicking on the first link below the table. The Read Processing Summary Table can be accessed by clicking on the second link below the table.

**More Information**

**Sample Information Table:**   
 1. **sample\_id:** unique identification code that was assigned to each sample.   
 2. **customer\_label:** sample name provided for the project and used in analyses.   
 3. **raw\_seq\_files:** names of the associated raw sequencing files for each sample (available from the raw data package download).   
 4. **Subgroup Columns:** group comparison information. If group comparison information was provided for the project, it is displayed in the remaining columns of the table.

**Read Processing Summary Table:**   
 1. **internal\_id:** unique identification code that was assigned to each sample.   
 2. **customer\_label:** sample name provided for the project and used in analyses.  
 3. **rawseqs(R1+R2):** number of raw sequences generated for each sample.  
 4. **trimmed\_seqs(R1+R2):** number of sequences retained after quality trimming.  
 5. **dada2\_infered:** number of sequences retained after DADA2 quality control trimming.  
 6. **chimera\_seqs:** number of chimeric sequences identified in the dada2\_infered sequences.  
 7. **chimera\_free\_seqs:** number of chimera-free sequences identified in the dada2\_infered sequences.  
 8. **unique\_seqs:** number of unique sequences identified in the chimera-free sequences.  
 9. **seqs(after\_size\_filtration):** number of chimera-free sequences that have also undergone further amplicon size filtration. This is the data that is finally analyzed for the rest of the report through QIIME.   
 10. **final\_unique\_seqs:** number of unique sequences identified in size-filtered chimera-free sequences.

| sample\_id | customer\_label | raw\_seq\_files |  |
| --- | --- | --- | --- |
| zr4724\_1V3V4 | Patient1 | zr4724\_1V3V4\_R1.fastq.gz;zr4724\_1V3V4\_R2.fastq.gz | NA |
| zr4724\_2V3V4 | Patient2 | zr4724\_2V3V4\_R1.fastq.gz;zr4724\_2V3V4\_R2.fastq.gz | NA |
| zr4724\_3V3V4 | Patient3 | zr4724\_3V3V4\_R1.fastq.gz;zr4724\_3V3V4\_R2.fastq.gz | NA |
| zr4724\_4V3V4 | Patient4 | zr4724\_4V3V4\_R1.fastq.gz;zr4724\_4V3V4\_R2.fastq.gz | NA |
| zr4724\_5V3V4 | Patient5 | zr4724\_5V3V4\_R1.fastq.gz;zr4724\_5V3V4\_R2.fastq.gz | NA |
| zr4724\_6V3V4 | Patient6 | zr4724\_6V3V4\_R1.fastq.gz;zr4724\_6V3V4\_R2.fastq.gz | NA |
| zr4724\_7V3V4 | Patient7 | zr4724\_7V3V4\_R1.fastq.gz;zr4724\_7V3V4\_R2.fastq.gz | NA |
| zr4724\_8V3V4 | Patient8 | zr4724\_8V3V4\_R1.fastq.gz;zr4724\_8V3V4\_R2.fastq.gz | NA |
| zr4724\_9V3V4 | Patient9 | zr4724\_9V3V4\_R1.fastq.gz;zr4724\_9V3V4\_R2.fastq.gz | NA |
| zr4724\_10V3V4 | Patient10 | zr4724\_10V3V4\_R1.fastq.gz;zr4724\_10V3V4\_R2.fastq.gz | NA |

**Complete Sample Information Table**

**Read Processing Summary Table**

# 2 Absolute Abundance

The plot below shows the absolute abundance of bacterial (16S) or fungal (ITS) DNA measured in the samples (based on the service requested). For analyses without group comparison, a histogram of gene copies per microliter in each sample is shown. For analyses with group comparison, a box-and-whisker plot of gene copies per microliter in each group is shown. The Absolute Abundance Table, which contains data for gene copies, calculated genome copies, and calculated amount of DNA can be accessed by clicking on the first link below the table. More information about the table can be found by clicking on the More Information button below.

**More Information**

**Absolute Abundance Table:**   
 1. **sample\_id:**unique identification code that was assigned to each sample.  
 2. **customer\_label:**sample name provided for the project and used in analyses.  
 3. **gene\_copies:** number of gene copies measured in one microliter of DNA sample.   
 4. **genome\_copies:** number of genome copies in one microliter of DNA sample calculated using an assumed number of four (4) 16S copies per genome or two hundred (200) ITS copies per genome.  
 5. **DNA\_ng:** amount of DNA in one microliter of DNA sample calculated using genome\_copies and an assumed genome size of 4.64 x 106 bp (Escherichia coli) for 16S or 1.20 x 107 bp (Saccharomyces cerevisiae) for ITS.

**Absolute Abundance Table**

# 3 Composition Barplots

Taxa composition plots illustrate the microbial composition at different taxonomy levels from phylum to species. The interactive figure below shows the microbial composition at species level. Additional composition barplots and abundance tables can be accessed by clicking on the link below the figure.

**Microbial Composition Barplots at All Phylogenetic Ranks**

# 4 Taxonomy Heatmaps

The taxonomy abundance heatmap with sample clustering is a quick way to help identify patterns of microbial distribution among samples. Heatmaps at different taxonomic levels and with or without sample clustering can be found by clicking the links below the figure.

**More Information**

The following heatmap shows the microbial composition of the samples at the species level with the top fifty most abundant species identified. Each row represents the abundance for each taxon, with the taxonomy ID shown on the right. Each column represents the abundance for each sample, with the sample ID shown at the bottom. If available, group information is indicated by the colored bar located on the top of each column. Hierarchical clustering was performed on samples based on Bray-Curtis dissimilarity. Hierarchical clustering was also performed on the taxa so that taxa with similar distributions are grouped together.

**Taxonomy Abundance Heatmap with Sample Clustering (Species)**

**Heatmaps with Sample Clustering:**   
   **Phylum**   **Class**     **Order**   **Family**    **Genus**   **Species**

**Heatmaps without Sample Clustering:**   
    **Phylum**   **Class**   **Order**   **Family**   **Genus**   **Species**

# 5 ASV Heatmaps

The amplicon sequence variant (ASV) abundance heatmap is built directly from the abundance of unique amplicon sequences inferred from raw sequencing data. Heatmaps with or without sample clustering can be found by clicking the links below the figure.

**More Information**

The following heatmap is created in a similar way as the taxa abundance heatmap described in Section 4. The DADA2 program used in the Zymo Research pipeline is able to differentiate a single nucleotide difference between two sequences. Sometimes, researchers may want to go beyond taxonomy assignment to look at the distribution of unique sequences. For example, two unique sequences different in 2 bp in the 16S V3-V4 region might be assigned to the same taxonomy; this might indicate the presence of two subspecies. This information can only be obtained by looking at the distribution of the abundance of unique sequences.

**Amplicon Sequence Variant (ASV) Heatmap with Clustering**

**Heatmap with Sample Clustering**

**Heatmap without Sample Clustering**

# 6 Alpha Diversity

Alpha diversity is a measurement of the microbial diversity of each sample. The plot below shows the number of observed species in the samples. For analyses without group comparison, a histogram of observed species in each sample is shown. For analyses with group comparison, a box-and-whisker plot of observed species in each group is shown. Alpha diversity graphs generated by other matrices can be found by clicking the last link

**More Information**

Normally, with deeper sequencing depth, the alpha diversity increases as more taxa at lower abundance are identified. Alpha diversity rarefraction graphs generated by other matrices can be found by clicking the link given below the figure.

**Alpha Diversity Histogram Plots**

**Alpha Diversity Rarefraction Plots**

# 7 Beta Diversity

Beta diversity is a measurement of microbial diversity differences between samples. The figure below is the 3-dimensional principle coordinate analysis (PCoA) plot created using the matrix of paired-wise distance between samples calculated by the Bray-Curtis dissimilarity using unique amplicon sequence variants (ASV). Interactive 3-dimensional plots of beta-diversity with different matrices can be accessed by clicking the links given below the figures.

**More Information**

Each dot on the beta diversity plot represents the whole microbial composition profile. Samples with similar microbial composition profiles are closer to each other, while samples with different profiles are farther away from each other.

**Beta Diversity 3D Emperor Plot View:**

**Bray-Curtis Plots:** **ASV**    **Genus**

**Weighted Unifrac Plots:** **ASV**

**Unweighted Unifrac Plots:** **ASV**

# 8 Taxa2ASV Decomposer

Taxa2ASV stands for taxonomy to amplicon sequence variations. In this analysis, a taxon of interest can be decomposed into its unique amplicon sequences to facilitate further analyses.

**More Information**

The information provided by the Taxa2ASV Decomposer analysis is useful for answering commonly-asked questions such as:  
   
1. Does the taxon of interest (e.g. Fusobacterium sp.) contain more than one unique sequence?   
2. How do the unique sequences assigned to this taxon compare to representatives in the reference database? What is sequence identity between the query sequence and the representative in the database?   
3. If there are more than one unique sequences, how similar/different are these sequences?   
4. If there are more than one unique sequences, how are these sequences distributed among samples?   
5. If I want to do my own analysis (e.g. Blast) with these unique sequences, where can I find them?   
The outputs of Taxa2SV decomposer are organized by taxonomy classifications (Family, Genus, and Species). This information is commonly used by researchers interested in a specific taxon. For each taxon, there are several outputs:   
1. **abun.plot.top12.pdf:** abundance distribution barplots. This file shows the distribution of the top twelve most abundant unique sequences as barplots.   
2. **annotation.txt:** taxonomy assignment table. This file lists all unique sequences, their assigned taxonomy, and the basis of the taxonomy assignment (sequence identity, hits in reference database,and the taxonomy of the hits in the reference database). This file allows you to re-examine taxonomy assignment of sequences of interest.   
3. **heatmap\_with\_clustering.pdf:** unique sequence abundance heatmap with sample clustering. This file views the distribution of unique sequences of a specific taxon across samples and groups. The unique sequences on the heatmaps are ordered by their phylogenetic distance as on the phylogenetic tree.   
4. **heatmap\_without\_clustering.pdf:** unique sequence abundance heatmap without sample clustering.   
5. **seq.counts.csv** sequence abundance distribution table in terms of number of sequences among samples.   
6. **seq.relative.abun.reorder.csv:** sequence abundance distribution table in terms of relative abundance (percentage) among samples.   
7. **seq.relative.abun.transpose.csv:** sequence abundance distribution table in terms of relative abundance (percentage) among samples, transposed.   
8. **seqs.align.html:** multiple sequence alignment of unique sequences involved. This file allows you to visualize the sequence variations of unique sequences identified in the taxon. The alignment was created using Muscle.   
9. **seqs.fna:** FASTA sequences of unique sequences involved. Sequences of interest can be extracted and used for additional analysis.   
10. **seqs.mltree.pdf:** neighbor-joined phylogenetic tree with maximum likelihood analysis. This allows you to identify groups of unique sequences, potentially discovering novel genera, species, or subspecies. If you are interested in a specific taxon, the best places to start are with the seq.relative.abun.reorder.csv file and the seqs.fna file. Together, these files will allow you to view the FASTA sequences of the unique sequences identified in the taxon and the relative abundance at which they were found in each sample.

**Taxa2ASV Decomposer Outputs Organized by:**  **Family**                 **Genus**
